# Acute kidney injury due to ammonium acid urate stones in a patient with adenovirus gastroenteritis: a case report

**DOI:** 10.1186/s12894-022-00954-4

**Published:** 2022-01-15

**Authors:** Hideki Ban, Kenichiro Miura, Rika Tomoeda, Katsuki Hirai, Motoshi Hattori

**Affiliations:** 1 Department of Pediatrics, Japanese Red Cross Kumamoto Hospital, 2-1-1 Nagamineminami, Higashi-ku, Kumamoto-shi, Kumamoto, 861-8520 Japan; 2 Department of Pediatric Nephrology, Tokyo Women’s Medical University, 8-1 Kawada-cho, Shinjuku-ku, Tokyo, 162-8666 Japan

**Keywords:** Acute kidney injury, Adenovirus gastroenteritis, Ammonium acid urate stone, Case report, Infant

## Abstract

**Background:**

Adenovirus gastroenteritis is a common cause of diarrhea and vomiting in infants, resulting in prerenal acute kidney injury (AKI). However, postrenal AKI due to urinary stones associated with adenovirus gastroenteritis is extremely rare. Here, we describe postrenal AKI due to obstructive ammonium acid urate stones associated with adenovirus gastroenteritis.

**Case presentation:**

A previously healthy 6-month-old boy had an 11-day history of severe diarrhea and a 5-day history of vomiting. His stool was positive for adenovirus antigens. We initiated fluid replacement therapy. On the second hospital day, he suddenly developed anuria. Abdominal computed tomography revealed bilateral hydronephrosis, left ureteral stones, and right bladder ureteral junction stones. Laboratory data showed that the creatinine level increased to 1.00 mg/dL. We diagnosed postrenal AKI due to obstructive bilateral urinary stones. Urination with stable urine volume resumed spontaneously after hydration. A few stones were found in the urine, which consisted of ammonium acid urate (> 98%). The serum creatinine level improved to 0.25 mg/dL. He was discharged nine days after admission.

**Conclusions:**

We suggest that adenovirus gastroenteritis be considered in pediatric patients with postrenal AKI due to urinary stones.

## Background

Acute gastroenteritis with viral infection is the most common cause of diarrhea and vomiting in infants, and it is the main cause of prerenal acute kidney injury (AKI). As reported by Fujinaga et al. [1], postrenal kidney injury may occur secondary to urinary stones associated with rotavirus gastroenteritis. Similar cases have been reported since 2005 [2, 3]. Postrenal AKI associated with rotavirus gastroenteritis is now widely recognized by pediatric nephrologists and urologists as a clinical pitfall. Postrenal AKI due to urinary stones associated with adenovirus gastroenteritis is extremely rare. Here, we describe a case of postrenal AKI due to obstructive ammonium acid urate stones associated with adenovirus gastroenteritis.

## Case presentation

**Fig. 1:**
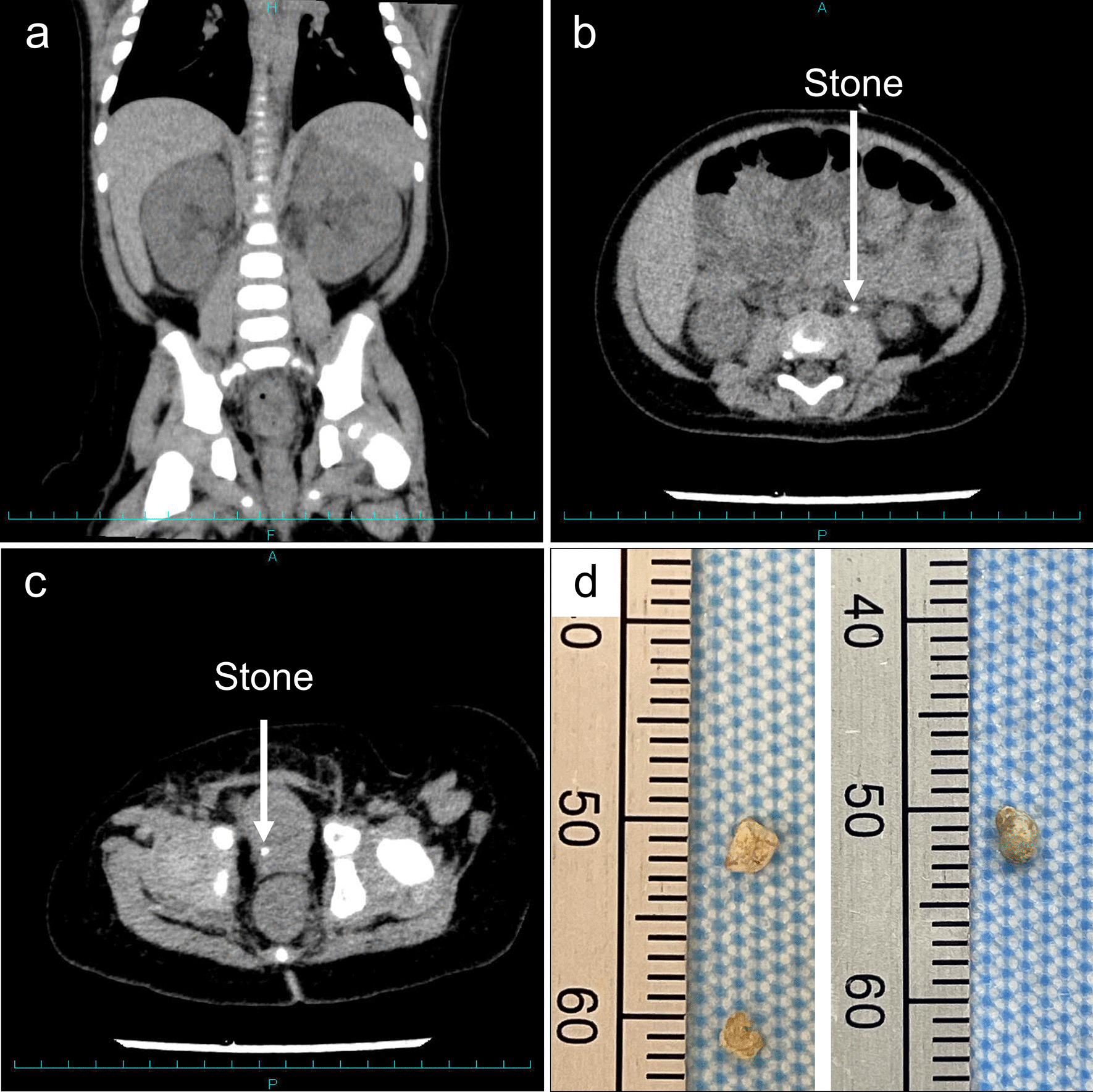
**a** Coronal computed tomography (CT) section reveals hydronephrosis. **b** Transverse CT section shows left ureteral stones (arrows). **c** Transverse CT section reveals right bladder ureteral junction stones (arrows). **d** A few stones with a size of 2–3 mm that were spontaneously excreted

This is the case of a previously healthy 6-month-old male infant with an 11-day history of severe diarrhea and a 5-day history of vomiting before admission at our hospital. He had no familial history of urolithiasis. Oral administration of the rotavirus vaccine had already been completed. He was initially treated at a local hospital where his laboratory findings revealed dehydration: blood urea nitrogen (BUN), 23.9 mg/dL; creatinine, 0.35 mg/dL; sodium, 135 mEq/L; and uric acid (UA), 13.0 mg/dL. He was transferred to our hospital after two days. On admission, his blood pressure was 107/79 mmHg; pulse was 135 beats per minute; and temperature was 37.8 °C. His body weight was 7.9 kg, 9.2% less than his pre-illness weight of 8.7 kg. His laboratory findings improved: BUN, 13.1 mg/dL; creatinine, 0.30 mg/dL; sodium, 150 mEq//L; UA, 5.6 mg/dL; pH 7.44; HCO_3_^−^, 21.1 mmol/L; and base excess, -2.2 mmol/L. Dipstick urinalysis revealed pH 6.0, specific gravity 1.020, hematuria (occult 3+), and proteinuria (2+). Fraction excretion of sodium and UA were 0.05% and 5.7%, respectively. His stool was positive for adenovirus and negative for rotavirus and norovirus antigens. As a treatment for adenovirus gastroenteritis, we initiated fluid replacement therapy. On the second hospital day, he suddenly developed anuria. An ultrasound (US) of the kidney showed a bilateral dilated pelvis (Society for Fetal Urology [SFU] grade I), although an obstructive lesion could not be identified. We continued fluid replacement therapy, assuming that the anuria was due to prerenal AKI, but it did not improve. Moreover, after fluid replacement therapy on the third hospital day, his body weight increased back to his pre-illness weight of 9.0 kg. US showed bilateral dilated pelvis (SFU grade II) and stone-like masses in the left ureter with no stones in the right urinary tract. We then performed abdominal computed tomography (CT), which revealed bilateral hydronephrosis (Fig. 1a), left ureteral stones (Fig. 1b), and right bladder ureteral junction stones (Fig. 1c). Laboratory data showed that the creatinine level increased to 1.00 mg/dL. Based on these findings, we diagnosed postrenal AKI due to obstructive bilateral urinary stones associated with adenovirus gastroenteritis. Because the urinary stones were obstructive, no pharmacologic interventions were initially planned and we intended to proceed with a percutaneous nephrostomy or transurethral ureterolithotripsy. However, during pre-surgical preparation, spontaneous urination with stable urine volume was observed and surgical plans were deferred. A few stones were noted in the urine (Fig. 1d), which mainly consisted of ammonium acid urate (> 98%). On the fourth hospital day (before the release of stone analysis results), the urine pH was 5.0. We initiated potassium citrate therapy, targeting a pH of 6.5–7.0. The following day, US confirmed resolution of the urinary stones and bilateral hydronephrosis; the serum creatinine level improved to 0.25 mg/dL. He was discharged nine days after admission.

## Discussion and conclusions

We experienced a case of adenovirus gastroenteritis postrenal AKI secondary to urinary stones and adenovirus infection. This case demonstrates the resolution of symptoms after medical management.

Over 60 human adenovirus serotypes have been described. Serotypes are further classified into seven species, A to G. As a urinary tract complication of adenovirus, adenovirus species B serotypes 11 and 21 have been associated with acute hemorrhagic cystitis in children [4, 5]. There are very few reports of urinary stones associated with adenovirus, and it is not speculated to be a direct effect of adenovirus infection on the urinary tract. In addition, adenovirus gastroenteritis is caused by the adenovirus species F serotypes 40 and 41 [5, 6], which is different from subgroup B, which has been associated with urinary tract complications. This further corroborates that adenovirus does not directly result in the formation of urinary stones.

Rotavirus, norovirus, and adenovirus are well-known causes of acute gastroenteritis in children. Among these viruses, there are some reports of urinary stones associated with rotavirus [2, 3, 7]. Ashida et al. [7] reported the clinical characteristics of patients with acute obstructive uropathy owing to bilateral urinary stones associated with rotavirus gastroenteritis. They revealed that (1) most patients were infants under 3 years of age, (2) most patients were males, (3) the mean period between the onset of oliguria and that of rotavirus gastroenteritis was 6.7 days (during the phase of recovery from dehydration), (4) most patients presented with hyperuricemia, (5) most patients had metabolic acidosis, (6) most patients presented with hyponatremia, and (7) most patients had stones that consisted mainly of ammonium acid urate. Our case was consistent with 5 of these 7 clinical features (No. 1, 2, 3, 4, and 7). In addition, although blood gas analysis at a local hospital was not performed, severe dehydration was observed, and metabolic acidosis might have been present. The clinical features of patients with urinary stones associated with rotavirus gastroenteritis and those with adenovirus gastroenteritis may be similar. However, there are very few reports of adenovirus gastroenteritis associated with urinary stones; therefore, this presumption is uncertain. It is necessary to conduct further analysis with a larger number of cases.

The frequency of ammonium acid urate stones is 0.38% [8], which is extremely rare among urinary tract stones. Soble et al. [9] reported that reduced urinary sodium excretion induces the binding of UA to large amounts of ammonia ions and promotes ammonium acid urate stones formation. Shirasu et al. [10] reported that prolonged metabolic acidosis (5–6 days) and dehydration were important factors in promoting the supersaturation of urinary ammonia urate. In our case, 12 days had passed from the onset of adenovirus gastroenteritis to the formation of urinary stones, and there was a 9.4% weight loss from his pre-illness body weight. There was probably prolonged metabolic acidosis and dehydration. Moreover, the fractional excretion of sodium at admission was 0.05%. We considered that these environments were conducive to ammonium acid urate stones formation.

The difference in disease frequency and the severity of gastroenteritis account for the fewer reports of urinary tract stones associated with adenovirus gastroenteritis compared to rotavirus gastroenteritis. Adenovirus is the cause of only 5–10% of acute gastroenteritis in young children, which is less than those due to rotavirus and norovirus [10]. Morita and Fujieda [11] reported that patients with rotavirus gastroenteritis experience high fever, high levels of hyperuricemia and acidosis, and a tendency toward high levels of urinary excretion of UA, β2-microglobulin, and N-Acetyl-beta-D-glucosaminidase compared with patients with norovirus gastroenteritis. These findings suggest that tissue damage caused by rotavirus is more severe than that caused by norovirus. Among pediatric patients with acute gastroenteritis, rotavirus gastroenteritis is the most important enteric infection causing urinary stones. Our report highlights that other etiologies, such as adenovirus gastroenteritis can cause urinary stones, suggesting that other viral causes of gastroenteritis should be considered. Previous reports of urinary stones associated with norovirus gastroenteritis [12, 13] support our proposal.

However, this case report had inherent limitations, such as the non-generalizability of our findings, inability to establish a cause-effect relationship, and risk of over-interpretation. Furthermore, as this case was of a previously healthy infant, no abdominal CT scans had been performed before the onset of adenovirus gastroenteritis. Therefore, it was not possible to definitively determine whether the adenovirus gastroenteritis was responsible for the urinary tract stone formation. Moreover, the time period of stone development was unclear; however, ammonia urate stones, which are the most frequently reported components of urinary tract stones associated with rotavirus gastroenteritis, were excreted in this case as well, suggesting that adenovirus gastroenteritis may have been involved in stone formation. Further, Ashida et al. [7] reported that the mean period between the onset of oliguria and that of rotavirus gastroenteritis was 6.7 days. Similar to our report, in the aforementioned work, the investigators could not perform CT examinations before stone formation; however, in general, urinary stones after viral enteritis may occur within a relatively short period. We searched PubMed for similar previous reports, but could not find any. We hope that, as a result of the information gathered in our report, a high index of suspicion for urinary stones will be given during the management of patients with adenovirus gastroenteritis, and that similar reports will increase as a result. Additional similar cases should be recruited for further investigation.

In conclusion, we suggest that adenovirus gastroenteritis be considered as a pathogenic agent resulting in postrenal AKI due to urinary stones.

## Data Availability

Records and data pertaining to this case are in the patient’s secure medical records in the Japanese Red Cross Kumamoto Hospital.

## Abbreviations

AKI: Acute kidney injury
BUN: Blood urea nitrogen
CT: Computed tomography
SFU: Society for Fetal Urology
UA: Uric acid
US: Ultrasound
